# Activated brain mast cells contribute to postoperative cognitive dysfunction by evoking microglia activation and neuronal apoptosis

**DOI:** 10.1186/s12974-016-0592-9

**Published:** 2016-05-31

**Authors:** Xiang Zhang, Hongquan Dong, Nana Li, Susu Zhang, Jie Sun, Shu Zhang, Yanning Qian

**Affiliations:** Department of Anesthesiology, the First Affiliated Hospital of Nanjing Medical University, Nanjing, Jiangsu 210029 People’s Republic of China; Clinical Research Center, the First Affiliated Hospital of Nanjing Medical University, 300 Guangzhou Road, Nanjing, Jiangsu 210029 People’s Republic of China

**Keywords:** Mast cells, Microglia activation, Neuronal apoptosis, Neuroinflammation, Postoperative cognitive dysfunction, Cromolyn

## Abstract

**Background:**

Neuroinflammation plays a key role in the occurrence and development of postoperative cognitive dysfunction (POCD). Microglia, the resident immune cells in the brain, has been increasingly recognized to contribute to neuroinflammation. Although brain mast cells (MCs) are the “first responder” in the brain injury rather than microglia, little is known about the functional aspects of MCs-microglia interactions.

**Methods:**

Male Sprague-Dawley (SD) rats were injected intracerebroventricular with MC stabilizer Cromolyn (100 μg/μl), MC stimulator C48/80 (1 μg/μl), or sterile saline 30 min before open tibial fracture surgery, and the levels of neuroinflammation and memory dysfunction were tested 1 and 3 days after surgery. In addition, the effect of activated MCs on microglia and neurons was determined in vitro.

**Results:**

Tibial fracture surgery induced MCs degranulation, microglia activation, and inflammatory factors production, which initiated the acute brain inflammatory response and neuronal death and exhibited cognitive deficit. Site-directed preinjection of the “MCs stabilizer” disodium cromoglycate (Cromolyn) inhibited this effect, including decrease of inflammatory cytokines, reduced MCs degranulation, microglia activation, neuronal death, and improved cognitive function 24 h after the surgery. In vitro study, we found that the conditioned medium from lipopolysaccharide (LPS)-stimulated mast cells line (P815) could induce primary microglia activation through mitogen-activated protein kinase (MAPK) pathway signaling and subsequent production of tumor necrosis factor-α (TNF-α) and interleukin-6 (IL-6). In addition, the activated P815 could directly induce neuronal apoptosis and synapse injury with microglia independently. Cromolyn could inhibit P815 activation following improved microglia activation and neuronal loss.

**Conclusions:**

These results implicate that activated MCs could trigger microglia activation and neuronal damage, resulting in central nervous system (CNS) inflammation, and communications of MCs with microglia and neuron could constitute a new and unique therapeutic target for CNS immune inflammation-related diseases.

## Background

Postoperative cognitive dysfunction (POCD), commonly observed in elderly patients, is characterized by impaired concentration, memory, and learning following surgery. POCD may be persistent over long durations of time or even progress into serious central nervous system diseases [1]. However, the pathophysiological mechanisms underlying POCD still remain unclear, and no disease-modifying or prophylactic therapies are currently available. Hence, unraveling the fundamental neuropathogenesis of POCD is an important challenge. Several studies have demonstrated that neuroinflammation caused by surgery is involved in the development of POCD [2, 3]. The initiation and propagation of neuroinflammation appear to rely very much on the interaction between microglia, immune cells, and neurons, with the microglia-immune cell connection being poorly explored thus far.

Microglia, the resident immune cells in the brain, play an important role in both initiation of protective immune responses and development of invasive inflammation during CNS disorders [4–7]. Increasing evidence has defined activated microglia as an important response of neuroinflammation [8]. When subjected to abnormal stimulation, microglia become gradually activated and produce numerous inflammatory mediators that may cause neuronal damage, starting a vicious cycle [9]. As such, inhibition of the exaggerated inflammatory response mediated by activated microglia cells may help to attenuate the severity of neurodegenerative diseases.

Although it is widely recognized that microglia activation contributes to neuroinflammation, proinflammatory signals released from other nonneuronal cells of immune origin like mast cells (MCs) are also critical players. MCs, best known for their role in allergic inflammation, also reside in the CNS, where they are often found along the blood vessels and leptomeninges [10, 11]. Under various types of stress, MCs serve as important sources of several mediators, including proteases and vasoactive amines such as GnRH [12], tryptase, and histamine [13], which have been reported to induce microglia activation and subsequent cytokine secretion in our previous studies [14, 15]. These inflammatory mediators released by MCs participate in several neuroinflammatory diseases [16]. Furthermore, meningeal MCs can recruit neutrophils and activated T cells to enter the brain by disrupting the blood-brain barrier [17]. Several molecular mechanisms for potential interactions between MCs and microglia have been determined in vitro [18]. However, the exact effect of MCs on microglia is still unclear. Given this background, we hypothesized that brain MCs, the first responders in the CNS after surgery, are responsible for inducing neuroinflammation and microglia activation, which may then cause cognitive dysfunction.

## Methods

### Animals

All experimental procedures involving animals were approved by IACUC (Institutional Animal Care and Use Committee of Nanjing Medical University). Male Sprague-Dawley rats weighted 200–250 g were used in this experiment procedure (*n* = 84). All rats were housed in groups of five animals per cage under specific pathogen-free conditions (ambient temperature, 22.0 ± 1.0 °C; humidity, 40 %) during breeding and the experiments. Food and water were available ad libitum. The experiments were approved by the Nanjing Medical University Animal Care and Use Committee.

### In vivo studies

#### Surgery and drug administration

The rats were randomly allocated to seven groups (groups A–G) with 12 rats in each group and investigators were blinded to the experimental treatment. Rats of group C and F were pretreated with MC stabilizer Cromolyn (100 μg/μl); groups D and G were pretreated with MC stimulator C48/80 (1 μg/μl); groups B and E were pretreated with sterile saline via intracerebroventricular injection (ICV) for 30 min after anesthesia and analgesia, while rats of group A was pretreated with sterile saline under conscious state. After 30 min, rats of groups E–G were treated with open tibial fracture surgery.

Following anesthesia, rats were placed in the stereotaxic apparatus (Stoelting Instruments, USA). Referring to the atlas of Paxinos and Watson (1982), guide cannulas (Plastic One) were inserted into the lateral ventricle (coordinates of 0.8 mm posterior, 1.5 mm lateral, and 3.7 mm ventral to the bregma) and secured to the skull with dental cement. The animals were allowed to recover in the animal facility for 14 days before use. Animals were handled daily to check the guide cannula and to familiarize them with the investigators. To investigate the exactly interactions between MC and microglia, we administered 2 μl of 1 μg/μl C48/80 (2.5 μg), 2 μl of 200 μg/μl Cromolyn, or 2 μl sterile saline ICV, respectively, 30 min before surgery.

Surgical animals underwent an open tibia fracture of the left hind paw with an intramedullary fixation under isoflurane anesthesia (2.1 % inspired concentration in 0.3 FiO2) as previously described [19]. Under full aseptic conditions, the left hind limb of surgical animals was meticulously shaved and disinfected with povidone iodine. Briefly, a middle incision was performed on the left hind paw followed by the insertion of a 20-G pin in the intramedullary canal; the periosteum was then stripped and osteotomy was performed. After producing the fracture, the wound was irrigated and the skin was sutured with 8/0 Prolene sutures. Temperature was monitored and maintained optimal with a warming pad throughout the surgical process. Analgesia (0.1 mg/kg buprenorphine) was given subcutaneously after anesthetic induction and before skin incision. Rats of group B were exposed to isoflurane anesthesia (2.1 % isoflurane in 0.3 FiO2) for 20 min in a temperature-controlled anesthetic chamber, shaved and received buprenorphine as described above only.

#### MCs staining and counting

Slides were stained in 0.05 % toluidine blue for hippocampus sections. A 1 % stock solution in 70 % ethanol was dissolved in 0.5 % NaCl solution (pH 2.2–2.3), and the slides were immersed 30 min in the above concentration. They were then washed twice in distilled water, dehydrated in a series of increasing concentrations of ethanol, followed by placement in butyl acetate ester. Samples were coverslipped using Eukitt® mounting medium and were allowed to dry overnight.

MC counting was always performed in a blinded fashion by an experimenter that was unaware of the sample identity. Criteria for degranulation included loss of purple staining, fuzzy appearance, distorted shape, or multiple granules visible in the vicinity of the cell. The entire surface area of the area CA1 of the hippocampus was scanned manually using a light microscope at ×200 magnification and MCs were calculated with the help of the Cell D software (Olympus).

In addition, immunohistochemistry was used to stain MCs with mast cell tryptase monoclonal antibody as follows.

### Microglia staining

In physical condition, most of microglia cells stay in a resting status, presented with obviously branch. Once been activated, microglia cells will become amoeboid, which had larger cell body, and poorly ramified short and thick processes. Immunohistochemistry was used to stain microglia cells with Iba1 polyclonal antibody as follows.

#### Immunohistochemistry

Rats were anesthetized by 5 % chloral hydrate (0.8 ml/100 g) and perfused first with 0.9 % saline and then with cold 4 % paraformaldehyde (0.1 M phosphate buffer, pH 7.4). The cerebral tissues were harvested, fixed with 4 % paraformaldehyde at 4 °C for 24 h. Brain sections (10-μm-thick) were prepared and immunohistochemistry was processed as follows. Endogenous peroxidase activity was blocked with 3 % H2O2 in PBS solution for 10 min. After incubation for 1 h in 10 % bovine serum albumin with 0.3 % Triton X-100 in 0.01 M phosphate buffer saline, the sections were then incubated with the mast cell tryptase monoclonal antibody (1:100; Abcam; USA), Iba1 polyclonal antibody (1:200; Wako; Japan), and caspase-3 monoclonal antibody (1:100; CST, USA) at 4 °C overnight and then incubated with secondary antibody for 2 h. Positive cells were visualized by adding DAB to the sections. For quantification [20], the studied tissue sections were selected with a 150-μm interval according to anatomical landmarks corresponding to bregma from bregma −2.8 to −3.8 mm of the rat brain (Paxinos and Watson, 1996). For each animal, 15 photographs from the CA1 area of three hippocampus sections and 15 photographs from the CA3 area of three hippocampus sections were captured using Leika 2500 (Leica Microsystems, Wetzlar, Germany) at ×200 magnification. The number of positive cells per photograph (0.74-mm^2^ frame) was obtained by using NIH Image J software (Bethesda, MD, USA), averaged, and converted to cells per square millimeter. Positive cell counting was performed in a blinded fashion by an experimenter that was unaware of the sample identity.

### Behavioral tests

#### Trace fear conditioning (TFC)

TFC is used to assess hippocampal-dependent memory in rodents as previously described [20–22]. Rats were trained to associate an environment (context) with a conditional stimulus (tone) and an unconditional stimulus (foot shock). The training consisted of placing the rat in the conditioning chamber and allowing exploration of the context for 100 s. Next, an auditory cue (80 dB, 5 kHz), the conditional stimulus, was presented for 20 s. A 2-s foot shock (0.8 mA), the unconditional stimulus, was administered after the termination of the tone. This procedure was repeated with an interval of 100 s, and the rats were removed from the chamber 30 s later. Contextual assessment was performed 24 or 72 h after surgery in the same chamber but with no cues (tone or shock). Freezing behavior, recognized as lack of movement, was recorded for 300 s by video and analyzed by software (Xeye Fcs, Beijing MacroAmbition S&T Development Co., Ltd., Beijing, China). A decrease in the percentage of time spent freezing indicated impairment of memory.

#### Y maze test

The Y maze consisted of three arms (regions I–III, 30 × 5 × 20 cm) that converged to an equilateral triangular central area (region 0). Each arm had a lamp at the distal end. A safe region was associated with the illumination, whereas the other regions featured electrical foot stimulation (40 ± 5 V). Each rat was first placed at the end of one arm (starting area chosen randomly) and allowed to move freely in the maze during a 3-min session without any stimulation to adapt to the environment. The test was then started and the illuminated arm (safe region) served as the new starting area. Furthermore, we changed the orientation of the safe and stimulation regions using a randomization method. The test was considered to be successful (learned) if the rat reached the safe region within 10 s. After each foot stimulation, we waited for the rat to reach the illuminated arm (the new starting area) before the next stimulation. If nine responses were correct in ten consecutive foot stimulations (9/10 standard), the rats were defined as having reached the learning criterion. The total number of stimulations to reach the criterion during training was recorded as the learning ability. All rats reached the learning criterion in the present study.

### In vitro studies

#### P815 cell culture

P815, a MCs line from mouse tumor cells, was provided by FuNing, PhD, Department of Immunology, Southern Medical University. The cells were incubated with DMEM medium containing inactivated 10 % fetal bovine serum and penicillin-streptomycin (0.6 × 10^5^ μl^−1^) at 37 °C and 5 % CO2 saturated humidity. The test was performed when the cells were in the logarithmic phase of growth.

#### Primary neuronal cultures

A pregnant C57BL/6 mouse (13–15 days) was killed by decapitation, fetal ones were isolated from the womb as previously described [23], and the skull was opened and the brain removed. After removal of the meninges from the whole brain, the regions of both hippocampus were harvested. The isolated samples of distinct brain regions were collected in separate Petri dishes in neurobasal medium containing 0.02 % BSA and transferred for tissue digestion into separate tubes containing the appropriate enzyme solutions. The issue was digested at 37 °C for 15 min in 0.25 % trypsin (2.5 % trypsin solution from Gibco was diluted 1:6 in PBS with EDTA). At the end of digestion, the tissue samples were centrifuged (1500 r.p.m. 5 min), and the supernatants were carefully removed. Cells were cultured with neurobasal medium supplemented with 2 % B27 (Gibco), 1 % glutamine 100 × (Gibco) and antibiotics. After 5–6 days, neurons with a better growth status were used in our study. The proportion of neurons in these cell cultures was more than 90 % as shown by immunohistochemistry using the anti-NSE antibody.

#### Microglia-enriched cultures

Microglial cells were obtained from the brains of newborn mice as described previously [24]. Briefly, mice were decapitated, their brains were dissected, and the meninges were carefully stripped off. Tissues were mechanically dissociated and resuspended in DMEM containing 10 % fetal calf serum, 100 U/ml penicillin, and 100 mg/ml streptomycin. Cultures were maintained at 37 °C in a humidified atmosphere of 5 % CO_2_/95 % air. After reaching a confluent monolayer of glial cells (10–14 days), microglia were separated from astrocytes by shaking off at 100 r.p.m for 1 h and replated on 24-well culture plates at a density of 10^5^ cells/cm^2^. The enriched microglia was >98 % pure as determined by Iba1.

#### Co-culture of microglia and P815 cells

After treatment with Cromolyn for 30 min, the P815 cells (1 × 10^6^ cells) were stimulated with lipopolysaccharide (LPS) (1 μg/ml) and cultured for up to 48 h. Primary microglia (1 × 10^6^ cells) were grown in 5 cm × 5 cm flasks until confluent and were treated with conditioned medium from P815 cells with different treatments for 48 h, which were incubated for an additional 24 h.

#### Co-culture of P815 cells and primary neurons

After treatment with Cromolyn for 30 min, the P815 cells (1 × 10^6^ cells) were stimulated with LPS (1 μg/ml) and cultured for up to 48 h. Primary neurons (1 × 10^6^ cells) were grown in 5 cm × 5 cm flasks until confluent and were treated with conditioned medium from P815 cells with different treatment for 48 h, which were incubated for an additional 24 h.

#### Immunofluorescence

To determine the activation of the microglia, the cells were fixed with 4 % paraformaldehyde for 30 min; nonspecific binding was blocked by incubating cells in a 5 % BSA (0.1 % Triton X-100) for 1 h at room temperature. The microglial cells were incubated with rabbit anti-Iba1 monoclonal antibody (1:200), and neurons were incubated with rabbit anti-Synapsin I antibody (SYP) (1:200) and rabbit anti-PSD95 (1:500) antibody in the blocking solution overnight at 4 °C. After three washes with PBS, cells were incubated with the FITC-conjugated goat anti-rabbit IgG (1:200) at 37 °C for 1 h and the nuclei were stained with DAPI. Fluorescent images were acquired using a confocal microscope.

### PCR

Total RNA from the hippocampus and primary microglia cell cultures was extracted with RNase (Takara), and reverse transcription was performed from 1 μg of total RNA for each sample using the Transcription First Strand cDNA Synthesis Kits (Roche) according to the manufacturer’s instructions. Real-time PCR amplification was performed using the STEP ONE Real-time PCR Detection System (Foster City, CA) with the SYBR Green master mix (Applied Biosystems, Foster City, CA) in a final volume of 10 μl that contained 1 μl cDNA template from each sample. Primers are listed as the following: rat CD86 forward, GACACCCACGGGATCAATTA reverse, AGGTTTCGGGTATCCTTGCT; rat CD32 forward, AGTTCGTTGCCGGTATTGAC reverse, TTCCCTGTGATCAGGGTTTC; rat IL-1β forward, ACTATGGCAACTGTCCCTGAAC reverse, GTGCTTGGGTCCTCATCCTG; rat CD206 forward, TGTGAGCAACCACTGGGTTA reverse, GTGCATGTTTGGTTTGCATC; rat SOCS3 forward, CCTCTGAGGTTCAGGAGCAG reverse, CGTTGACAGTCTTCCGACAA; rat GAPGH forward, GGGTGTGAACCACGAGAAAT reverse, CCACAGTCTTCTGAGTGGCA; mouse CD86 forward, GACCGTTGTGTGTGTTCTGG reverse, GATGAGCAGCATCCAAGGA; mouse CD32 forward, GAAACCCTGATCACAGGGAA reverse, TTTGGCAGCTTCTTCCAGAT; mouse IL-1β forward, TTGACGGACCCCAAAAGAT reverse, GAAGCTGGATGCTCTCATCTG; mouse CD206 forward, CAAGGAAGGTTGGCATTTGT reverse, CCTTTCAGTCCTTTGCAAGC; mouse SOCS3 forward, TGCAGGAGAGCGGATTCTAC reverse, TGACGCTCAACGTGAAGAAG; mouse GAPDH forward, AACTTTGGCATTGTGGAAGG reverse, GGATGCAGGGATGATGTTCT. The cycling conditions were 95 °C for 30 s followed by 40 cycles of 95 °C for 5 s and 60 °C for 30 s. The relative mRNA values were normalized to the GAPDH gene control values and calculated using the comparative cycle threshold (ΔΔCt) method.

### TNF-α and IL-6 assay

The content of tumor necrosis factor-α (TNF-α) and interleukin-6 (IL-6) in rat hippocampus tissue extracts and the amount of TNF-α and IL-6 in the culture medium were measured with a commercial ELISA kit from R&D Systems.

### Western blotting

Ipsilateral hippocampus tissues, microglia cells, and neurons were homogenized in RIPA lysis buffer (Biyuntian, Shanghai, China), which contained 50 mM Tris (pH 7.4), 150 mM NaCl, 1 % Triton X-100, 1 % sodium deoxycholate, 0.1 % sodium dodecyl sulfate (SDS), sodium orthovanadate, sodium fluoride, EDTA, leupeptin, etc. The homogenates were centrifuged at 12,000*g* for 20 min (4 °C), and the supernatants were then harvested as cytosolic fractions for immune blot analysis. After incubation for 20 min on ice, lysate was centrifuged and protein concentration was determined by the BCA kit. Proteins (50 μg) were denatured with SDS sample buffer and separated by 10 % SDS-polyacrylamide gel electrophoresis. Proteins were transferred to PVDF membranes (Millipore) by using a Bio-Rad miniprotein-III wet transfer unit. The membranes were incubated with 5 % BSA dissolved in Tris-buffered saline with Tween 20 (TBST) (pH 7.5, 10 mM Tris–HCl, 150 mM NaCl, and 0.1 % Tween 20) at room temperature for 1 h. This was followed by incubating the membranes with different antibodies overnight at 4 °C. The following primary antibodies were used: rabbit monoclonal anti-c-Jun N-terminal kinase (JNK), phospho-JNK, extracellular regulated protein kinases (ERK), phospho-ERK, AKT, phospho-AKT, Bax, Bcl-2 (1:1000), rabbit polyclonal anti-SYP and PSD95, and mouse monoclonal anti-matrix metalloproteinase-9 (MMP-9). After adding the goat-anti-rabbit or goat-anti-mouse secondary antibody (1:5000) for 1 h, the protein bands on the membranes were detected with an enhanced chemiluminescence kit.

### Transferase dUTP nick end labeling (TUNEL) assay

Terminal deoxynucleotidyl TUNEL assay, a method for detecting DNA fragmentation, was used to measure neuronal apoptosis. The one-step TUNEL apoptosis assay kit is obtained from KeyGEN BioTECH (KGA7074). In brief, after respective treatment, cells plated on slides were fixed with 4 % formaldehyde for 30 min in room temperature. After washed three times by PBS, slides were incubated with 1 % Triton X-100 for 5 min and then incubated with FITC-conjunctive fluorescein-12-dUTP for 30 min in 37 °C. The nuclei were stained with DAPI. Fluorescent images were acquired using a confocal microscope.

### Statistical analysis

All values are means ± SD. The significance of the difference between control and samples treated with various drugs was determined by one-way ANOVA followed by the post hoc least significant difference test. Differences were considered significant at *P* < 0.05.

## Results

1. Cromolyn inhibited surgery-induced MCs degranulation in the hippocampus
  To examine whether MCs are involved in surgery-induced neuroinflammation, we first quantified brain MCs in toluidine blue (TB) and MC tryptase stained sections of the hippocampus at 1 and 3 days after surgery. As shown in Fig. 1a, b, surgery led to a significant increase in MCs number in area CA1 of the hippocampus and was significantly greater than the counts in the control group at 1 and 3 days after surgery. This effect was inhibited by directed site injection in the right ICV of MC stabilizer Cromolyn (200 μg, 2.5 μl). In addition, directed site injection of the mast cell degranulator C48/80 (2.5 μg) alone also promoted the increasing number of MCs obviously at 1 day, similar to the effect induced by surgery. Furthermore, site injection of C48/80 (2.5 μg) 30 min before surgery caused a synergistic increase in the number of MCs in the hippocampus. And site injection of Cromolyn (200 μg) alone had no effect on MCs in the brain. These results suggest that surgery can induce MC degranulation in the hippocampus.

**Fig. 1:**
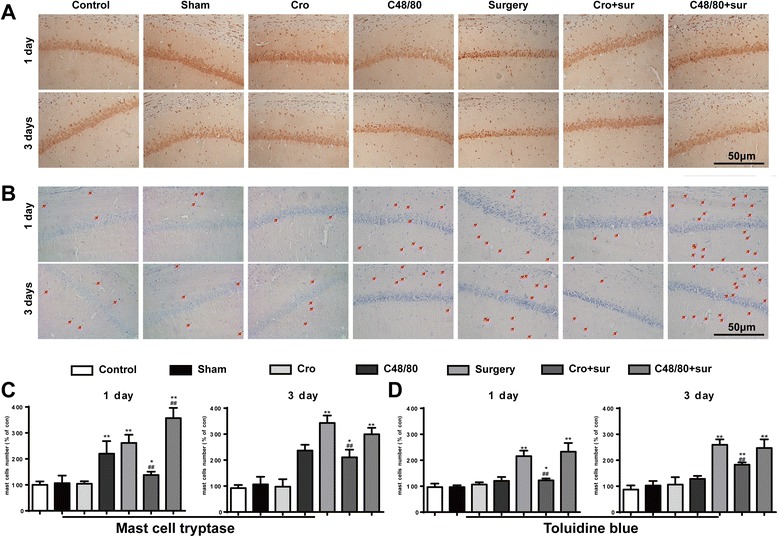
Cromolyn inhibited surgery-induced MC degranulation in the hippocampus. **a** Immunostaining was used to detect mast cell tryptase in area CA1 of the hippocampus. *Scale bar* 50 μm. **b** Mast cells were stained with toluidine blue (TB) in area CA1 of the hippocampus. *Scale bar* 50 μm. **c** Quantification of Tryptase-positive cells in area CA1 of the hippocampus. **d** Quantification of mast cells stained with TB. ^*^ *P* < 0.05, ^**^ *P* < 0.01 vs*.* control group. ^#^ *P* < 0.05, ^##^ *P* < 0.01 vs*.* surgery group. Data are presented as the mean ± SD (*n* = 6)
2. Cromolyn inhibited microglia activation in the hippocampus induced by the tibial surgery
  In order to explore the effects of activated MCs on microglia activation, immunostaining was used to detect Iba1, a marker for microglia. Surgery led to notable increases in microglia activation in the hippocampus at 1 and 3 days, as indicated by a large number of Iba1-ir cells, which had larger cell body, and poorly ramified short and thick processes (Fig. 2a, b). The effect could once again be prevented through pretreatment with a MC stabilizer and aggravated by pretreatment with a MC degranulator. Treatment with the MC degranulator once again led to a similar readout as surgery. To further observe the effect of activated MCs on phenotype change of microglia in the hippocampus, the phenotype markers of microglia in area CA1 of the hippocampus at 1 day were tested. Surgery increased the expression of M1 markers (CD86, CD32, IL-1β) and immunomodulatory M2b marker (SOCS3) caused a decrease in the expression of M2a-repair/regeneration marker (CD206), which instead of the activation of microglia (Fig. 2c–g). These results suggest that stabilization of MC can inhibit the activation of microglia.

**Fig. 2:**
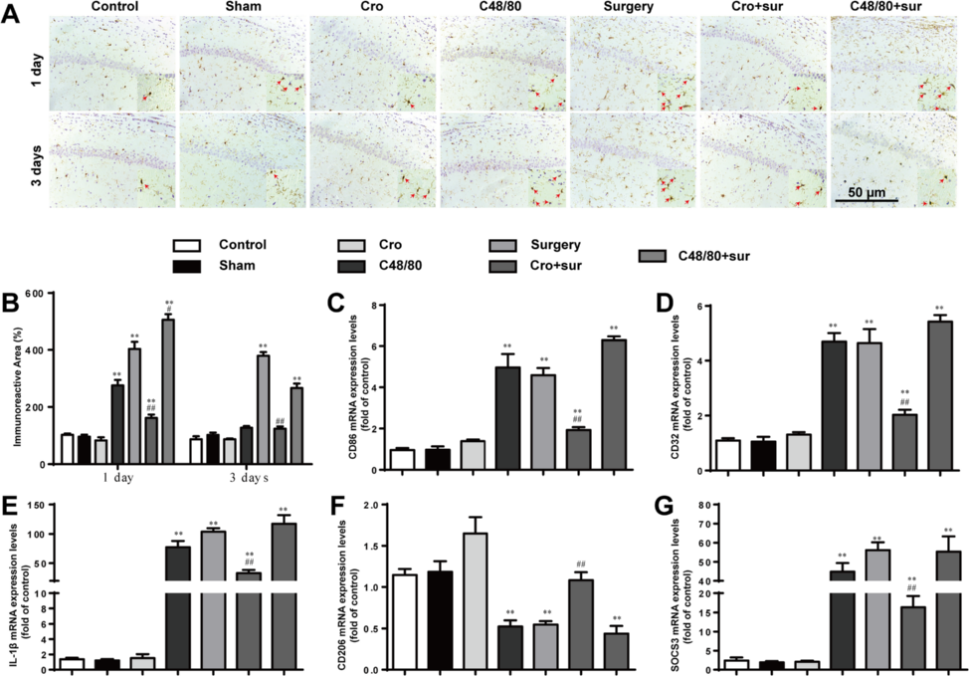
Cromolyn inhibited microglia activation in the hippocampus induced by the tibial surgery. **a** Immunostaining was used to detect Iba1 in area CA1 of the hippocampus, markers for microglia. *Scale bar* 50 μm. **b** Quantification of Iba1-positive cells in area CA1 of the hippocampus. **c**–**e** Expression levels of M1 phenotype markers (CD86, CD32, and IL-1β). **f** Expression levels of CD206, markers for M2a phenotype. **g** Expression levels of SOCS3, markers for M2b phenotype. ^*^ *P* < 0.05, ^**^ *P* < 0.01 vs*.* control group. ^#^ *P* < 0.05, ^##^ *P* < 0.01 vs*.* surgery group. Data are presented as the mean ± SD (*n* = 6)
3. Cromolyn inhibited surgery-induced and neuronal apoptosis in the hippocampus and ameliorated the cognitive decline
  To further confirm whether activated mast cells participate in cognitive dysfunction, we tested for neuronal apoptosis with caspase-3 immunostaining and assessed learning and memory with behavioral tests. To further examine neuronal injury, we then explored the expression levels of amyloid precursor protein (APP), PSD95, and Bax/Bcl-2. Surgery could induce dramatic increases in caspase-3 expression in the hippocampus at 1 and 3 days postoperation, and Cromolyn (200 μg) capably protected against neuronal injury, with the same response with APP, PSD95, and Bax/Bcl-2 expression (Fig. 3a, b, e, and f). Meanwhile, injection of Cromolyn alone did not change the freezing time and number of learning trials. However, compared against controls, the rats exposed to surgery exhibited a significant reduction in cognitive function at 1 and 3 days. Notably, treatment with Cromolyn significantly improved the freezing behavior and the number of learning trials at 1 and 3 days (Fig. 3c, d). These results suggest that Cromolyn may help protect against surgery-induced cognitive dysfunction.

**Fig. 3:**
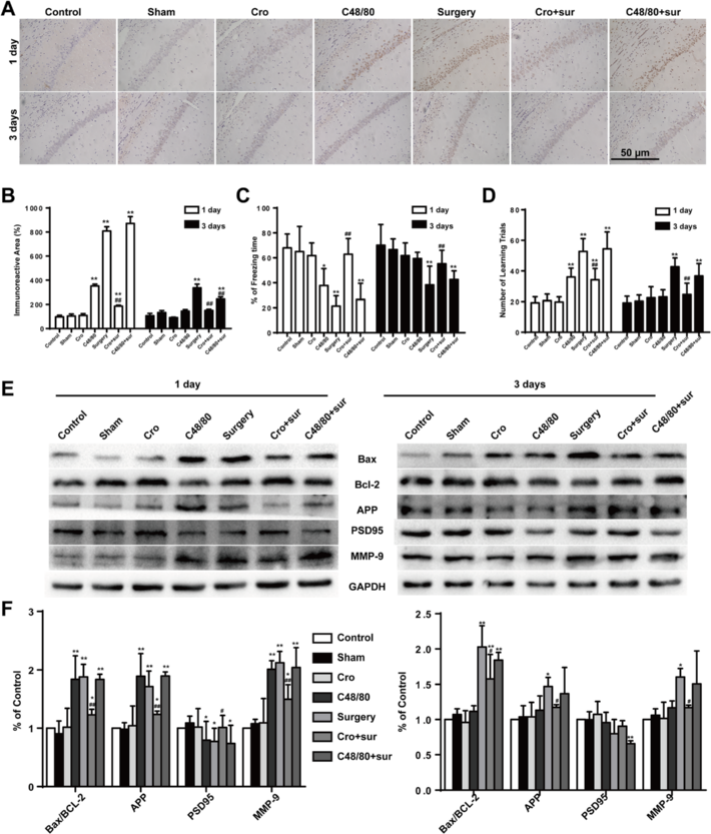
Cromolyn inhibited surgery-induced and neuronal apoptosis in the hippocampus and ameliorated the cognitive decline. **a** Immunostaining was used to detect caspase-3 in area CA1 of the hippocampus. *Scale bar* 50 μm. **b** Quantification of caspase-3-positive cells in area CA1 of the hippocampus (*n* = 6). The freezing time in trace fear conditioning test (**c**) and the number of learning trials in Y Maze test (**d**) were recorded to analyze the cognitive changes (*n* = 10). **e** The expression of Bax, Bcl-2, APP, PSD95, and MMP-9 was detected by Western blotting using specific antibodies in the hippocampus of rats. Each blot is representative of three experiments. **f** Expression of Bax was quantified and normalized to Bcl-2 levels, and expression of APP, PSD95, and MMP-9 were quantified and normalized to GAPDH levels. Each value was then expressed relative to the control, which was set to 1 (*n* = 5). ^*^ *P* < 0.05, ^**^ *P* < 0.01 vs*.* control group. ^#^ *P* < 0.05, ^##^ *P* < 0.01 vs*.* surgery group. Data are presented as the mean ± SD
4. Cromolyn inhibited surgery-induced mitogen-activated protein kinase (MAPK) activation and cytokines secretion in the hippocampus
  Since microglia-mediated neuroinflammation is mainly due to the excessive secretion of proinflammatory factors from activated microglia and their downstream signaling cascades, the levels of proinflammatory factors TNF-α and IL-6 were detected by ELISA. Surgery induced significant increases in TNF-α and IL-6 production, and site injection of Cromolyn (200 μg) could inhibit the inflammatory response (Fig. 4c, d). To confirm the signaling pathways involved in neuroinflammation, MAPK-related proteins were tested with Western blot. Surgery led to the rapid phosphorylation of ERK and JNK in the hippocampus, a change that can be prevented by Cromolyn (200 μg) pretreatment (Fig. 4a, b). In addition, site injection of Cromolyn (200 μg) alone had no effect on cytokine production in the hippocampus. Furthermore, pretreatment with C48/80 (2.5 μg) could exacerbate the inflammatory response of CNS induced by surgery. These results suggest that stabilization of MCs can inhibit the production of proinflammatory factors in the hippocampus, and MAPK signaling pathways were involved.

**Fig. 4:**
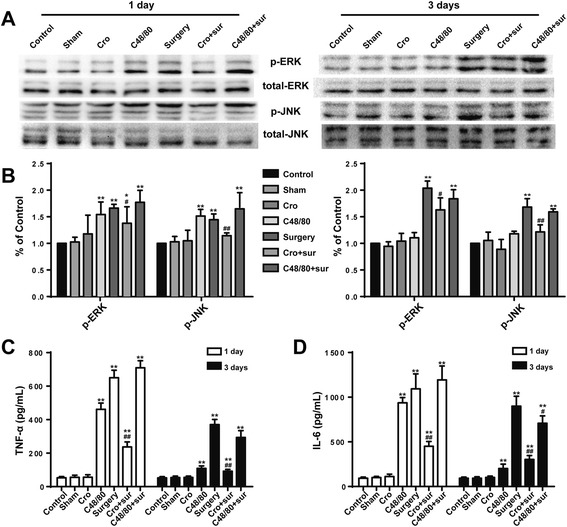
Cromolyn inhibited surgery-induced MAPK activation and cytokine secretion in the hippocampus. **a** The activated levels of ERK and JNK in the area CA1 of the hippocampus, which was assessed by increased phosphorylation of tyrosine residues of these kinases, were detected by Western blotting using specific antibodies. Each blot is representative of three experiments. **b** Phosphorylated levels of ERK and JNK were quantified and normalized to total levels. Each value was then expressed relative to the control, which was set to 1. **c**–**d** The levels of proinflammatory factors TNF-α and IL-6 were detected by ELISA. ^*^ *P* < 0.05, ^**^ *P* < 0.01 vs*.* control group. ^#^ *P* < 0.05, ^##^ *P* < 0.01 vs*.* surgery group. Data are presented as the mean ± SD (*n* = 5)
5. Activated P815 cells increased microglia activation
  Given that our previous results clearly indicate a correlation between MCs and neuroinflammation caused by surgery, we then sought to clarify the importance of this correlation. Since microglia activation is an early sign that triggers neuronal death in neurodegenerative disorders, we explored the effect of activated MCs on microglia in vitro. Microglia activation was detected via immunofluorescent staining for Iba1. The transfer of medium from P815 cells stimulated with LPS could induce a significant increase in microglia activation, an effect inhibited by the additional treatment of cromolyn. With the same effect of LPS (1 μg/ml) alone, incubated with CM from P815 with LPS-stimulation (48 h) for 24 h could induce microglia activation obviously. Pretreatment with Cromolyn (10 μg/ml) for 30 min suspended the activated effect of MCs on microglia (Fig. 5a). To further confirm the effect of P815 on phenotype change of primary microglia, the phenotype markers of microglia were tested. LPS (1 μg/ml) increased the expression of M1 markers (CD86, CD32, IL-1β) and immunomodulatory M2b marker(SOCS3), caused a decrease in the expression of M2a-repair/regeneration marker (CD206) [25], both directly and through P815 cells. Pretreatment with Cromolyn (10 μg/ml) for 30 min can inhibit microglia activation effectively (Fig. 5b–f). These results indicated that activated secreted products from P815 could induce microglia activation and change the phenotypes of microglia towards M1/2b.

**Fig. 5:**
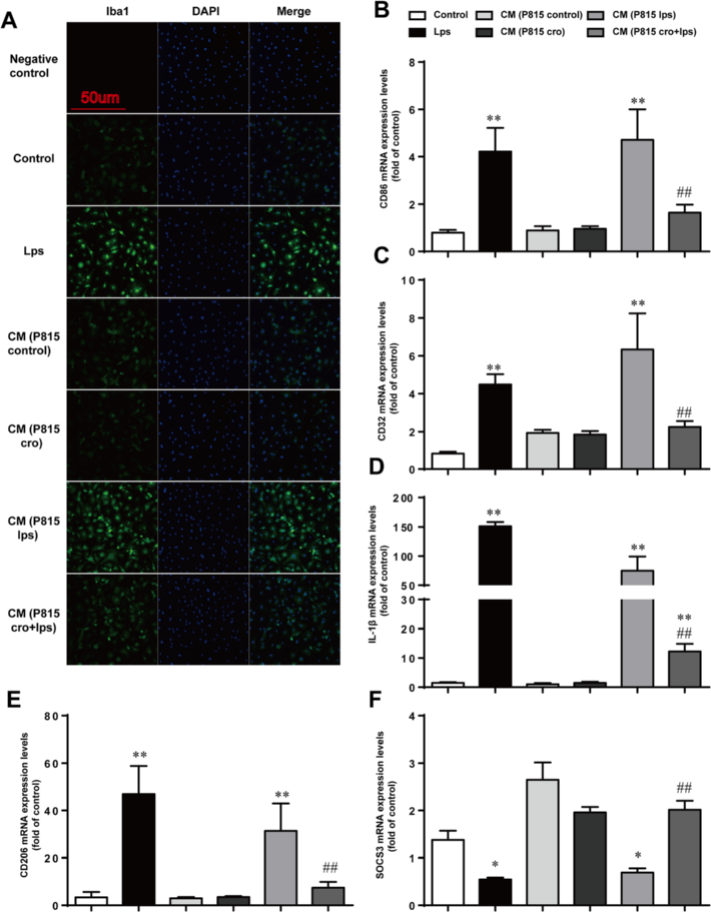
Activated P815 cells increased microglia activation. **a** The cells were stained with Iba1 antibody, and upregulated Iba1-immunopositive expression (*green*) on the activated microglia was observed using confocal scanning. The *blue* staining represents DAPI. Scale bar 50 μm was used to detect Iba1. **b**–**d** Expression levels of M1 phenotype markers (CD86, CD32, and IL-1β). **e** Expression levels of CD206, markers for M2a phenotype. **f** Expression levels of SOCS3, markers for M2b phenotype. ^*^ *P* < 0.05, ^**^ *P* < 0.01 vs*.* control group. ^#^ *P* < 0.05, ^##^ *P* < 0.01 vs*.* CM (P815 lps) group. Data are presented as the mean ± SD (*n* = 5)
6. MAPK signaling pathways were involved in microglia activation and cytokine production
  Since changes in MAPK signaling pathways were observed in the MC-induced microglia activation in vivo, we then investigated these signaling pathways in vitro for further verification. LPS stimulation (48 h) of microglia in vitro stimulated a higher phosphorylation of ERK and JNK, which was ameliorated by Cromolyn (10 μg/ml) pretreatment (Fig. 6a, b). To observe the inflammatory effects of MC microglia co-culture, proinflammatory factors TNF-α and IL-6 in the supernatant were detected with ELISA. As shown in Fig. 6c, d, with the same effect of LPS alone, cytokine production was also sharply upregulated by CM from P815 with LPS stimulation, and P815 with Cromolyn (10 μg/ml) pretreatment could improve the inflammatory response. Collectively from this data, we concluded that MAPK signaling pathways were involved in MC-induced microglia activation.

**Fig. 6:**
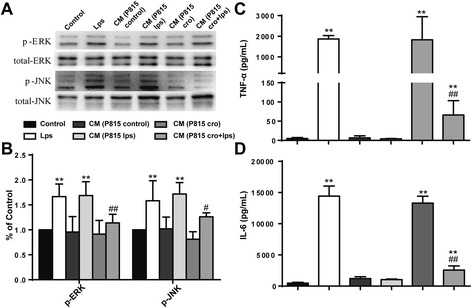
MAPK signaling pathways were involved in microglia activation and the following cytokine production. **a** The activated levels of ERK and JNK, which was assessed by increased phosphorylation of tyrosine residues of these kinases, was detected by Western blotting using specific antibodies. Each blot is representative of three experiments. **b** Phosphorylated levels of ERK and JNK were quantified and normalized to total levels. Each value was then expressed relative to the control, which was set to 1. **c**–**d** The levels of proinflammatory factors TNF-α and IL-6 were detected by ELISA. ^*^ *P* < 0.05, ^**^ *P* < 0.01 vs*.* control group. ^#^ *P* < 0.05, ^##^ *P* < 0.01 vs*.* CM (P815 lps) group. Data are presented as the mean ± SD (*n* = 5)
7. Activated P815 cells induced neuronal apoptosis
  To explore the direct effect of activated MCs on neurons, we tested neuronal apoptosis and synaptic changes. With the same effect of LPS (1 μg/ml) alone, incubated with CM from P815 with LPS, stimulation (48 h) for 24 h could induce neuronal loss. Results from TUNEL test showed that CM from P815 with LPS stimulation could induce neuronal apoptosis (Fig. 7a), presented with the SYP and PSD95 changes (Fig. 7b–d). The above results indicated that activated MCs could induce neuronal apoptosis directly.

**Fig. 7:**
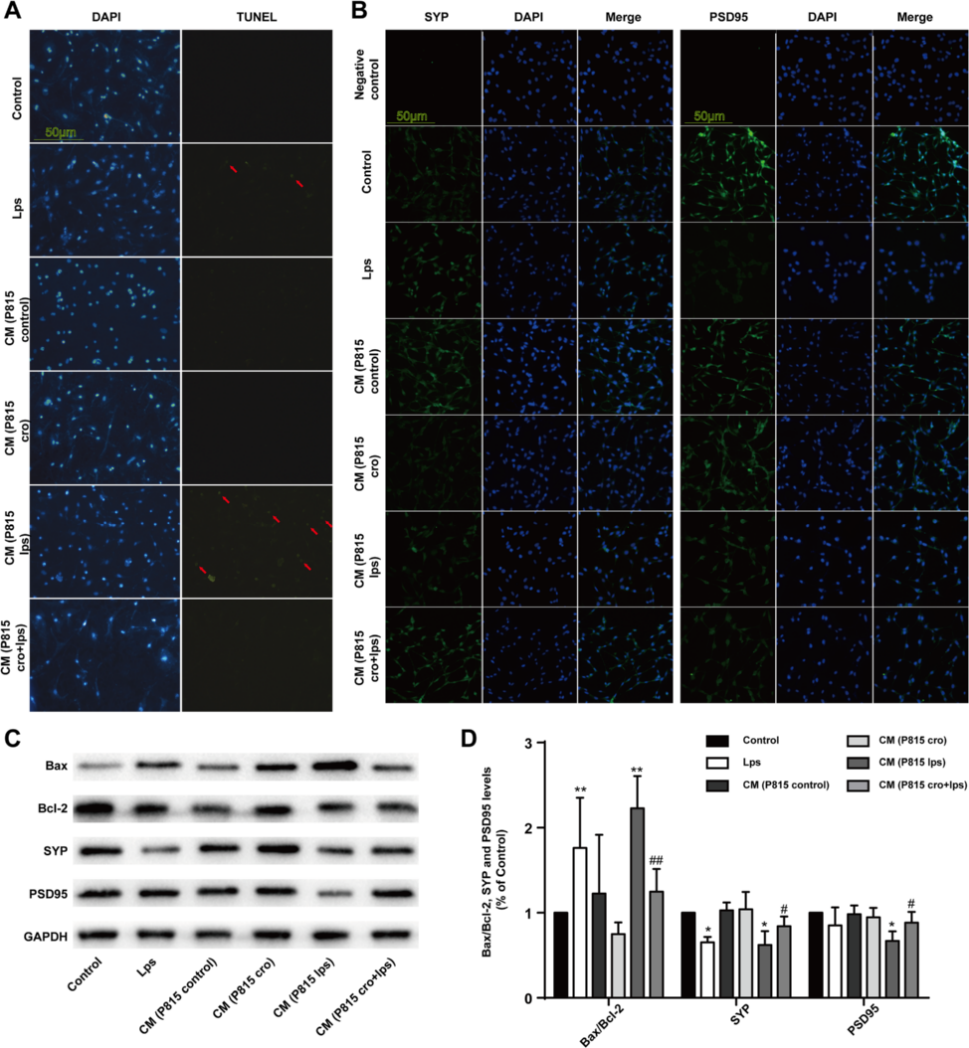
Activated P815 cells induced neuronal apoptosis directly. **a** Neuronal apoptosis was tested with TUNEL test. **b** The cells were stained with SYP and PSD95 antibody and upregulated SYP, and PSD95-immunopositive expression (*green*) on the activated microglia was observed using confocal scanning, respectively. The *blue* staining represents DAPI. Scale bar 50 μm was used to detect Iba1. **c** The expression of Bax, Bcl-2, SYP, and PSD95 was detected by Western blotting using specific antibodies in the hippocampus of rats. Each blot is representative of three experiments. **d** Expression of Bax was quantified and normalized to Bcl-2 levels. Expression of SYP and PSD95 were quantified and normalized to GAPDH levels. Each value was then expressed relative to the control, which was set to 1. ^*^ *P* < 0.05, ^**^ *P* < 0.01 vs*.* control group. ^#^ *P* < 0.05, ^##^ *P* < 0.01 vs*.* CM (P815 lps) group. Data are presented as the mean ± SD (*n* = 5)

## Discussion

Pathological mechanisms that regulate neuroinflammation have not been well characterized, even though neuroinflammation may provoke many clinically important phenomena, such as the occurrence and development of cognitive dysfunction after peripheral surgery. Previous authors have raised the concern that important therapeutic targets against neuorinflammation were being missed by exploring microglia and MCs in isolation from each other [18]. In this study, we explored the effect of MCs on microglia activation and its role in POCD. We demonstrate that activated brain MCs are able to activate microglia and induce neuronal apoptosis, aggravating neuroinflammation. We show that this activation can occur as a result of peripheral surgery and that cognitive function strongly correlates with MC activation.

Cognitive dysfunction after peripheral surgery, whether in the form of temporary delirium or POCD, has been associated with long-term adverse outcomes including low quality of life, heavier burden of economy, and an increasing mortality rate [26, 27]. In rodents, POCD symptoms can be modeled with deficits in exploratory behavior and spatial-based working memory. TFC is widely used to assess learning and memory in rodents [28]. In the present study, we found that predirected site injection in the right ICV with MC stabilizer Cromolyn improved learning and memory of SD rats by increasing the rate of freezing time both 1 and 3 days after the tibial surgery. Directed site injection of C48/80 alone, which is MC degranulator, could also induce cognitive deficiency, as the same effect with peripheral surgery at 1 day after surgery. These results were then confirmed via the Y maze test, one of the most widely used paradigms for detecting spatial-based working memory in rodents [29]. It is very sensitive for measuring early-onset postoperative cognitive deficit and is usually used to assess hippocampus-dependent working memory [30, 31]. Meanwhile, hippocampal neurogenesis-related cognition is found to decline by using electric shocks Y maze [32]. In our study, we also used this type of Y maze test to evaluate the spatial-based working memory 1 and 3 days after the tibial fracture surgery, and the assessment was approximately the same as TFC. Thus, we considered that activated MCs may participate in the cognitive deficiency after the tibial surgery. The study by Nautiyal et al. implicates brain mast cells in the modulation of anxiety-like behavior and provides evidence for the behavioral importance of neuroimmune links [33]. Approximately one third of patients with mastocytosis display neuropsychological symptoms [34]. Masitinib, an inhibitor of the survival, migration, and activity of MCs, can improve the cognitive decline in ADs [35]. All of those showed the relationship between MCs and cognitive decline.

Inflammation is fundamentally protective in restoring injury and initiating the healing process, but destructive when prolonged to override the bounds of physiological. Increasing evidence recognize neuroinflammation as a key element in the pathobiology of neurodegenerative diseases [36–38]. As the sensor cells for disturbed brain tissue homeostasis, particularly in response to neuron injury, microglia cells release proinflammatory molecules that play key roles in neurodegenerative diseases [39]. Activation of microglia has been demonstrated to be an early sign that often precedes and triggers neuroinflammation and neuronal death in neurodegenerative diseases [40, 41]. Our previous studies found that microglia activation participate in the surgery-induced neuroinflammation and cognitive decline through TLR4/MyD88 signaling, which can be improved by preintraperitoneal injection of lithium for continuous 6 days before splenectomy [42]. As the similar results shown in this study, microglia cells were activated significantly in surgery group after the tibial surgery. At the same time, it also showed with neuronal apoptosis and cytokine secretion simultaneously in CA1 region of the hippocampus. MAPK signaling pathway, which is an important regulator of inflammation, was involved. Microglia cells mediate multiple facets of neuroinflammation, including cytotoxicity, repair, regeneration, and immunosuppression and have the ability to acquire diverse activation states or phenotypes [25]. We found that surgery increased expression of cytotoxic M1 markers (CD86, CD32, and IL-1β) and immunomodulatory M2b markers (SOCS3). Meanwhile, surgery caused a decrease in the expression of M2a-repair/regeneration markers (CD206). Within the aged brain, microglia cells are primed and easily produce a more violent response to inflammatory stimulation [43]. Microgliosis, which is defined as an increased number of microglia, is an important response of neuroinflammation [8]. Therefore, the inhibition of microglial activation may offer prospective clinical therapeutic benefits for neuroinflammation-related neurodegenerative disorders.

Prototypical proinflammatory stimuli (LPS, IL-1β, and TNFa) have been shown to increase microglia activation and cytokine production [25]. However, the factors contribute to the overactivation of microglia are still largely covered. Notably, microglia cells also respond to proinflammatory substances released from other immune cells such as MCs. The corresponding receptors of tryptase and histamine have been found on the surface of microglia in our previous studies [14, 15]. MCs are found in most tissues, including CNS, where they are often found adjacent to blood vessels and nerves [44] and can traverse the blood-brain barriers in pathological conditions [45]. Emerging evidence of MCs-microglia interaction opens new insights for therapies targeting neuroinflammation by modulating activation of MCs that may control the overactivation of microglia. Jin et al. demonstrated that MC activation is the “first responder” in this type of injury, preceding microglia activation [46]. Consistent with it, in the present vivo study, we found that surgery could activate not only MCs but also microglia at 1 but 3 days that was ameliorated by Cromolyn. The study by Schemann et al. demonstrated that the MC degranulator C48/80 may directly activate neurons, which can be preserved by histamine H1 and H_2_ antagonists [47]. To further ensure the effect of MCs on microglia directly, we co-cultured P815 cells and primary microglia in vitro. We found that LPS-stimulated P815 cells can activate microglia and induce TNF-α and IL-6 release. Pretreatment with “Mast cell stabilizer” Cromolyn (10 μg/ml) for 30 min could inhibit the phenomenon. However, the P815 cells without LPS stimulation had no effect on microglia activation. MAPK signaling pathway was involved. These results further confirm that activated MCs can induce microglia activation and cytokine release.

Although the functional relationship between MCs and neurons in CNS is not yet well clarified, communication between MCs and peripheral nerves has been examined and may be helpful to inform CNS roles [48]. Cell adhesion molecule-1d expressed by mature hippocampal neurons also is found to be the isoform that MCs most strongly adhere to in vitro, as part of a direct, contact-based signaling [49]. Mediators released from MCs, like nerve growth factor and ATP, are required for neuronal survival and physical function [50, 51]. All the above reveal the close communication between MCs and neurons, which may be a fundamental element for the physical neuronal function. But in pathological conditions, whether degranulation of MCs can result in neuronal apoptosis is still unknown. In the present vivo study, we found that the activation of MCs can induce neuronal apoptosis and neuro-synapsis injury. To eliminate the effect of microglia, we co-cultured P815 cells and primary neuron in vitro in order to ensure that the effect was indeed a direct one. The results revealed that activated MCs can induce neuronal apoptosis directly, without microglia intermediaries. We therefore anticipate that surgery-induced MC degranulation can result in hippocampal neuronal damage both via, and independent of, microglia activation.

## Conclusions

In summary, the present study identifies that peripheral surgery induce MCs degranulation, following by microglia activation and neuronal apoptosis, resulting in neuroinflammation and cognitive dysfunction, which can be inhibited by the “MCs stabilizer” Cromolyn. In vitro study further confirms that only the activated MCs can induce microglia activation, and MAPK pathways are involved. In addition, activated MCs could directly induce neuronal apoptosis with microglia independently These results implicate that activated MCs could not only trigger microglia activation but also induce neuronal damage directly, resulting in neuroinflammation, and interactions between MCs, microglia, and neuron could constitute a new and unique therapeutic target for neuroinflammation-related diseases.

## Highlights

- Surgery can induce mast cells degranulation, microglia activation, and neuronal apoptosis.
- “Mast cell stabilizer” Cromolyn can inhibit surgery-induced microglia activation and neuronal apoptosis.
- Activated mast cells can evoke microglia activation and neuroinflammation.
- Activated mast cells can induce neuronal apoptosis directly.

## Abbreviations

APP, amyloid precursor protein; CNS, central nervous system; Cromolyn, disodium cromoglycate; ERK, extracellular regulated protein kinases; ICV, intracerebroventricular injection; IL-6, interleukin-6; JNK, c-Jun N-terminal kinase; LPS, lipopolysaccharide; MAPK, mitogen-activated protein kinase; MCs, mast cells; MMP-9, matrix metalloproteinase-9; POCD, postoperative cognitive dysfunction; SDS, sodium dodecyl sulfate; SYP, Synapsin I antibody; TB, toluidine blue; TBST, Tris-buffered saline with Tween 20; TFC, trace fear conditioning; TNF-α, tumor necrosis factor-α.
